# Assessment of Internal Jugular Vein Size in Healthy Subjects with Magnetic Resonance and Semiautomatic Processing

**DOI:** 10.1155/2016/9717210

**Published:** 2016-02-29

**Authors:** M. M. Laganà, L. Pelizzari, E. Scaccianoce, O. Dipasquale, C. Ricci, F. Baglio, P. Cecconi, G. Baselli

**Affiliations:** ^1^IRCCS, Fondazione Don Carlo Gnocchi ONLUS, Via Capecelatro 66, 20148 Milan, Italy; ^2^Department of Electronics, Information and Bioengineering, Politecnico di Milano, Piazza Leonardo da Vinci 32, 20133 Milan, Italy

## Abstract

*Background and Objectives*. The hypothesized link between extracranial venous abnormalities and some neurological disorders awoke interest in the investigation of the internal jugular veins (IJVs). However, different IJV cross-sectional area (CSA) values are currently reported in literature. In this study, we introduced a semiautomatic method to measure and normalize the CSA and the degree of circularity (Circ) of IJVs along their whole length.* Methods*. Thirty-six healthy subjects (31.22 ± 9.29 years) were recruited and the 2D time-of-flight magnetic resonance venography was acquired with a 1.5 T Siemens scanner. The IJV were segmented on an axial slice, the contours were propagated in 3D. Then, IJV CSA and Circ were computed between the first and the seventh cervical levels (C1–C7) and normalized among subjects. Inter- and intrarater repeatability were assessed.* Results*. IJV CSA and Circ were significantly different among cervical levels (*p* < 0.001). A trend for side difference was observed for CSA (larger right IJV, *p* = 0.06), but not for Circ (*p* = 0.5). Excellent inter- and intrarater repeatability was obtained for all the measures.* Conclusion*. This study proposed a reliable semiautomatic method able to measure the IJV area and shape along C1–C7, and suitable for defining the normality thresholds for future clinical studies.

## 1. Introduction

The cerebrospinal venous system has been the focus of many studies in the last few years, because of the hypothesized involvement of insufficient extracranial venous drainage in central nervous system disorders such as multiple sclerosis, normal-pressure hydrocephalus, and transient monocular blindness [1–4]. An insufficiency in venous blood drainage can be due to the presence of single or multiple stenosis on the main routes of cerebrospinal venous system [5].

The internal jugular veins (IJVs), together with the vertebral veins, constitute the predominant extracranial pathways for the cerebral venous drainage [5, 6]. The cerebrospinal venous system is characterized by a great anatomical variability and complex hemodynamics, which is not entirely comprehended [7]. Since numerous variability factors must be considered, the investigation of IJVs anatomy and hemodynamics is not trivial. Indeed, IJVs present great intersubject variations, and within the same subject, the right internal jugular vein (IJVr) lumen is usually greater than the left internal jugular vein (IJVl) one [8]. Moreover, since veins are compliant vessels, their cross-sectional area (CSA) depends also on the subject position, head rotation, breathing, and cardiac function [5, 7]. Therefore, the quantitative IJV morphological analysis* in vivo* is still an open issue, despite the clinical need to determine what can be considered as a significant IJV CSA reduction with respect to a physiological range.

The normal IJV CSAs values reported by literature are difficult to compare since measurements were performed at different levels, corresponding to few anatomical landmarks (e.g., cricoid cartilage level, thyroid gland midlevel, C2-C3 level, C5-C6 level, and C7-T1 level), which cannot be considered representative of the whole IJV. Different values were obtained through autopsy [9], or* in vivo* with imaging techniques such as ultrasound [10], magnetic resonance (MR) [11–13], and computed tomography [14].

The routine* in vivo* IJV inspection is currently based on ultrasounds and/or MR. Although color Doppler ultrasonography is generally used for screening, it is operator-dependent and limited in its field of view. On the other hand, magnetic resonance venography (MRV) allows an operator-independent acquisition and a 3D reconstruction that completely depicts IJVs and its collaterals along their whole length, from the base of the skull to the subclavian vein. MRV can be performed with an endovenous contrast agent (magnetic resonance angiography and venography, MRAV), or using an endogenous source of contrast (time-of-flight magnetic resonance venography, TOF MRV). Specifically, the latter approach is a noninvasive technique, which enhances the blood that flows through a slice. Although an international consensus regarding the available imaging modalities for the IJV investigation was recently published [7], the problem of defining a threshold to discriminate a physiological IJV from a pathological one is not solved yet.

The aim of this study was to introduce a semiautomatic approach for measuring the IJV CSA* in vivo* along the cervical levels from C1 to C7 and for their normalization in order to define a normality range. In order to achieve this goal, healthy subjects' IJVs were imaged through TOF MRV and segmented. Furthermore, the degree of circularity (Circ) was introduced to obtain also quantitative information about IJV shape.

## 2. Materials and Methods

### 2.1. Subjects and Image Acquisition

Thirty-nine healthy volunteers with no history of medical, vascular, or neurological illnesses were enrolled for this study. None of the recruited subjects received monetary compensation for participating in the study.

MRI data was acquired on a 1.5 Tesla Siemens Magnetom Avanto at IRCCS, Don Carlo Gnocchi Foundation, Milan, Italy.

The acquisition protocol consisted of the following sequences. Firstly, brain dual-echo turbo spin echo (repetition time (TR) = 2,650 ms; echo times (TE) = 28/113 ms; echo train length = 5; 50 contiguous 2.5 mm thick axial slices; 1 mm^2^ in-plane resolution), and fluid attenuated inversion recovery sequence (FLAIR) (TR = 8002 ms; TE = 128 ms; inversion time (TI) = 2000 ms; 48 contiguous 3 mm thick slices) were acquired to exclude subjects with evidence of focal white matter pathology and any anatomical abnormality. Then, 2D TOF MR venography covering the whole neck, with a saturation band positioned caudal to the 128 axial slices (TR = 26 ms, TE = 7.2 ms, flip angle = 70°, in-plane resolution = 0.5 × 0.5 mm^2^, FOV = 256 × 192 mm^2^, slice thickness = 3 mm, and distance factor between subsequent slices = −20%) was acquired. During the acquisition, the subjects were supine on the scanner table and were asked to breathe quietly and regularly.

An expert radiologist evaluated the TOF images and excluded a subject from the following morphological analysis if the IJV borders were not clearly enhanced in two consecutive slices or more, either for banding artifacts, or for the absence of one or more IJV tracts. In the latter case, ultrasound was also used to confirm IJV agenesis, according to the multimodal approach recommended by Zivadinov and colleagues [7].

The study protocol was in accordance with the principles of the Helsinki Declaration and it was approved by the “Don Carlo Gnocchi Foundation” ethics committee, Milan, Italy. A written informed consent was obtained from all the study participants.

### 2.2. Image Processing

The IJVl and IJVr of all the subjects who met the defined inclusion criteria were segmented by a trained operator. The segmentation was performed with Jim 6.0 software package (Xinapse Systems, UK, http://www.xinapse.com/) on all the slices between C1 and C7, with the following steps. The C1 and C7 levels were identified on the TOF sagittal view and the image contrast was set to enhance IJV. Then, each IJV was semiautomatically segmented on a single slice with the edge detection and contour following algorithm [15]. Edge seeking and 3D propagation modes were set, so the IJV edge was automatically propagated on the other slices. Finally, the obtained 3D regions of interest (ROIs) were checked slice by slice and manually cleaned if needed, in order to exclude all the structures that had been wrongly recognized as IJV by the automatic propagation. The time required for segmentation (including the cleaning) was measured.

The IJV CSAs and perimeters (*P*) were automatically measured for each segmented slice. Circularity (Circ) [16] was computed as

(1) $$\begin{array}{c} Circ=\frac{4\pi CSA}{P^{2}}. \end{array}$$

In order to make data comparable among subjects of different C1–C7 length, the CSA- and Circ-to-slice curves were interpolated using Matlab (MATLAB Release 2013a, The MathWorks, Inc., Natick, Massachusetts, USA), setting the distance between C1 and C7 to the median C1–C7 length evaluated across subjects.

The subject with the median C1–C7 length was identified, and the samples corresponding to each cervical level (C1, C2, C3, C4, C5, C6, and C7) were defined on his/her TOF images.

### 2.3. Statistical Analysis and Repeatability

Group parametric descriptive statistics were calculated for CSA and Circ. According to Kolmogorov-Smirnov test results, we reported the means and standard deviations for the variables with a normal distribution, otherwise we reported the median and the 5th, 25th, 75th, and 95th percentiles. The IJV CSA and Circ differences among the seven cervical levels were tested taking into account the side, using a two-factor repeated measures ANOVA. The alpha level of 0.05 was considered significant.

**Figure 1:**
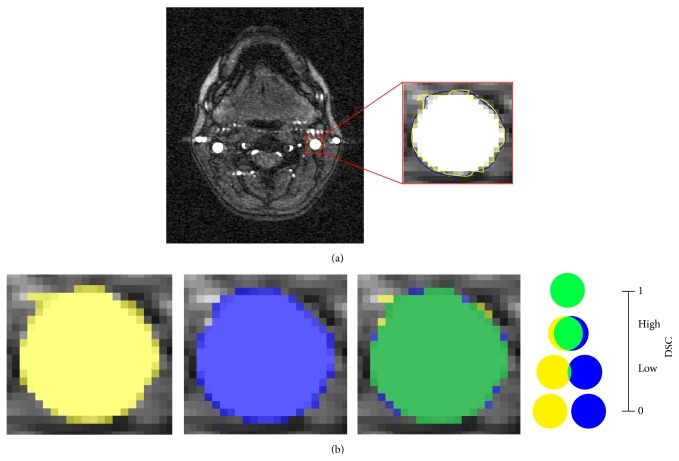
(a) shows the segmentation IJVl on a TOF MR image by two different operators (operator 1, yellow line; operator 2, blue line). (b) shows a graphical representation of Dice similarity coefficient (DSC): how it is computed and how it can be interpreted. Yellow area corresponds to the ROI identified by operator 1 (ROI1), blue area corresponds to the ROI identified by the operator 2 (ROI2), and green area is the overlapping region of the yellow and blue ROIs (ROI1∩ROI2). The DSC is computed as 2*∗*CSA_ROI1∩ROI2_/(CSA_ROI1_ + CSA_ROI2_). A schematic representation of different DSC values is reported on the right side of panel (b): from 0 (no overlap of the two areas) to 1 (perfect overlap of the two areas). Note that for equal cross-sectional areas, the other reliability indexes (*ρ*c and ICC) are perfect (equal to 1); instead, DSC can change, depending on the overlap of the two ROIs.

The intraoperator reliability of the measures was assessed on ten IJVrs and ten IJVls: the main operator repeated the segmentation twice. A second operator performed the same segmentation without being aware of the main operator's IJV borders, for assessing the interoperator reliability. Lin's concordance correlation coefficient (*ρ*c) [17] and the intraclass correlation coefficient (ICC) [18] were computed. In addition, Dice similarity coefficient (DSC) was calculated for each slice, in order to assess the agreement on the position of the segmented IJVs (Figure 1).

The strength of the agreement according to *ρ*c and DSC was classified as moderate between 0.60 and 0.80, substantial between 0.80 and 0.90, and almost perfect higher than 0.90. No systematic differences were assumed for ICC higher than 0.90.

Statistical analyses were performed using SPSS (version 21; IBM Corp., Armonk, NY, USA).

## 3. Results

### 3.1. Subjects

All the acquired TOF images were evaluated to be of good quality by an expert radiologist. Although none of the recruited subjects had anatomical abnormalities at DE and FLAIR images inspection, three subjects out of 39 (males, 24 ± 2.3 years) were excluded due to unilateral IJV agenesis, confirmed with ultrasound. The remaining 36 subjects were 13 males and 23 females, with average (standard deviation) age of 31.22 (9.29) years. The body mass index in this group ranged from 18.80 to 24.70, with a median value of 22.00; thus, every subject had a normal weight for his/her height.

### 3.2. Segmentation

The edge seeking algorithm applied on the TOF-MRV images of all the included subjects allowed segmenting the IJVs satisfactorily along the seven cervical levels, even though all the generated 3D ROIs needed to be cleaned manually by the operator. The cleaning mainly consisted in deleting ROIs generated in non-IJV areas, and, to a lesser extent, in modifying IJV borders. The IJV segmentation took between 8 and 15 (median = 10) minutes per IJV.

### 3.3. Anatomical Measurements

The C1–C7 segment had a median (range) length of 120 mm (95.48–144.00 mm). All the CSA- and Circ-to slice curves were resampled to 50 samples (median C1–C7 length divided by slice spacing = 120 mm/2.4 mm).

**Figure 2:**
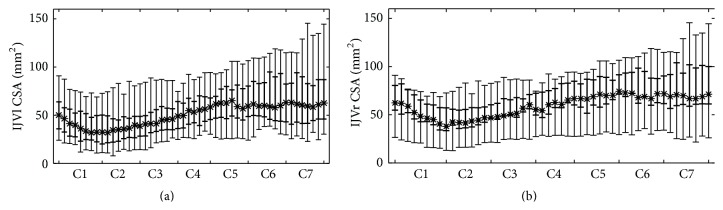
Descriptive statistics of IJVl CSA (a) and IJVr CSA (b) displayed along C1 and C7 cervical levels. The median (asterisks), the 5th–95th percentiles (bars), and the 25th–75th percentiles (bold bars) are represented.

Group average CSA and Circ values for each level are reported in Table 1. The IJVl and IJVr CSAs across the 50 samples are shown in Figures 2(a) and 2(b), respectively.

The left-right comparison showed a trend for higher right compared to left IJV CSA (Table 1; mean IJVl CSA = 53.49 mm^2^, mean IJVr CSA = 63.26 mm^2^; *p* = 0.06) aggregating the data of all the cervical segments. Six out of 36 subjects (16.7%) were IJVr dominant (IJVr CSA higher than twice the IJVl CSA); three out of 36 subjects (8.3%) were IJVl dominant.

**Figure 3:**
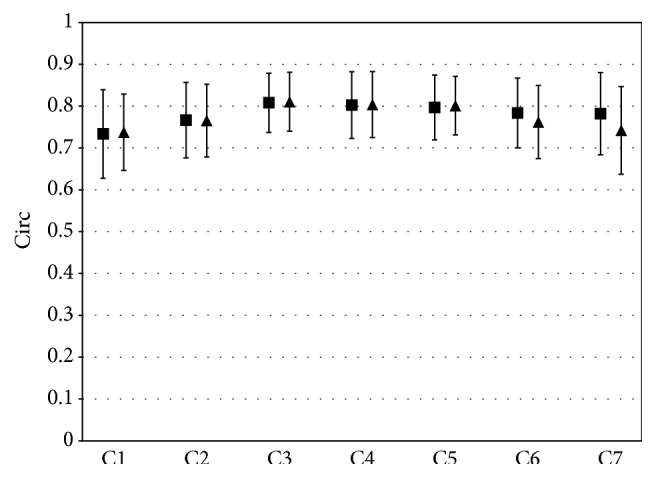
Group average IJVl (squares) and IJVr (triangles) Circ for each cervical level. Bars represent standard deviations.

Conversely to CSA, no trend for side difference was observed for Circ (*p* value = 0.5, Figure 3).

The IJV CSA and Circ were significantly different among the various cervical levels (*p* < 0.001), without interaction with the side.

### 3.4. Repeatability

The intrarater and interrater variability tests showed that there was an almost perfect agreement (*ρ*c > 0.95) and no systematic differences (ICC > 0.90) in the CSA measures (Table 2).

Furthermore, considering the DSC calculated on all the segmented slices for each considered subject, high values (median DSC > 0.90) were generally observed both for inter and intrarater comparisons (Table 3).

## 4. Discussion

In this study, we implemented a semiautomatic method to measure IJV size and shape along the cervical levels from C1 to C7 on TOF images. TOF MRI sequence is particularly suitable for acquiring wide groups of healthy controls due to the absence of endovenous contrast agent.

By adopting this semiautomatic method, the operator intervention is limited to the definition of the slices corresponding to the upper C1 and the lower C7 margins, to IJV border selection in one slice and to ROI cleaning. All these steps require few minutes and the “normalization” along the vessel length allows aggregating subjects of different height and C1–C7 length, without the need of identifying several anatomical landmarks on each subject's TOF image. Therefore, differently from the current methodology which consists in measuring the CSA at few points corresponding to specific anatomical levels (e.g., thyroid cartilage or C2-C3 or C5-C6 levels), this approach provides measures at many points along the IJV, with the a priori definition of just two anatomical landmarks (C1 and C7). The importance of measuring the CSA for the whole IJV length was confirmed by the results in our group of healthy controls, since the IJV size was significantly different among the different cervical levels. The presented method allows the generation of normality values in a group of healthy subjects for all the seven cervical levels and it could also be used for the evaluation of any subject IJV morphology with respect to the group ones.

The level of agreement obtained with the inter- and intrarater variability tests and the high DSC values highlighted the excellent reliability of our method. In order to achieve repeatable results, the most critical part is the manual initialization of the segmentation. Depending on IJV CSA visualization, it is related to the venous velocity and to the image contrast adjustment. The former is an intrinsic source of variability for the TOF MRI, because the faster the blood flows, the higher its enhancement is; the latter can be improved with training.

Comparing the CSA values reported by literature with the corresponding level of our data showed that the absolute IJV CSA values obtained with our work were lower than those presented by previous studies [10, 11, 14]. This discrepancy could be due to the differences in the imaging techniques, for example, ultrasound [10], MR [11], and computed tomography [14]. The IJV CSA underestimation by TOF with respect to contrast enhanced MRAV was observed in a study of Haacke's group [11]. The sources of the differences are multiple: firstly, the TOF-MRV signal depends on flow velocity and thus regions characterized by a slow flow, such as the IJV walls, could be missed [19]. Second, slow flow could be confused with stenosis or agenesis. However, in our study we carefully excluded this error by confirming the IJV agenesis with ultrasound evaluation. Third, the TOF are generally acquired on the axial slice with a higher resolution compared to the sagittal or coronal MRAV. The differences between our results and the 2D TOF-MRV results obtained by the previously cited work [11] instead could be ascribed to different acquisition parameters: we used a 1.5 T scanner while Rahman and colleagues acquired with a 3 T one, and the axial resolution was not the same (0.5 × 0.5 mm^2^ versus 0.63 × 0.63 mm^2^). A partial volume effect in the IJV borders, due to the inclusion in the same pixel of venous blood or its surrounding tissue, can alter the segmentation and the CSA area. Another intrinsic source of variation is the different sample group, with demographic differences such as the age and the country of origin of the subjects.

Interestingly, our group CSA standard deviations were high, specifically between 34.2% and 53% of the average values, which confirms the high physiologic venous variability. However, the high reliability of our semiautomatic segmentation approach limited the analytic variability; thus, the statistical consistency was improved with respect to previous studies [11, 14, 20]. The distal parts (C6-C7 levels) of our IJV segmentation had the highest standard deviations, probably because of breathing artifacts, which can alter the border identification.

Notably, even though our mean CSA is lower compared to literature, we obtained some clinically relevant results in accordance with previous studies. Firstly, the average of the CSA fifth percentiles along C1–C7, defining the lower limit of normality for our group of healthy subjects (Figure 2), is similar to the clinical CSA threshold used to classify an IJV as stenotic using ultrasound [1, 10], that is, 30 mm^2^ for the right and 29 mm^2^ for the left side. Secondly, our group of healthy subjects showed a trend for larger right compared to left IJVs, similarly to previous studies that assessed IJVs asymmetry [8, 9, 14]. In the third place, despite the side differences, IJVl and IJVr CSA showed a similar trend across levels (see Figure 1), decreasing from C1 to C2 and increasing from C3 to C7. The same trend can be observed in the results of Jurkiewicz and colleagues [13] for children and adolescents, even if all their subjects were younger than ours (age < 18 years).

In the context of combined studies on neurodegeneration and vascular alterations, the proposed IJV segmentation method can provide reproducible IJV CSA measures that can be used to assess the correlation with clinical or MRI-derived neurodegenerative indices. Indeed, as we showed by modeling the cerebrospinal venous system and by simulating progressive IJV stenoses [21], an IJV patency decrement theoretically increases the intracranial pressure and thus could lead to neurodegenerative effects. Furthermore, the proposed normalization approach can be used for case-control studies.

Another novelty introduced with this study is the circularity index Circ for venous anatomical assessment. This shape index has been previously applied in arterial studies only [16], but it could also be promising for quantitatively evaluating the veins. Indeed, currently the IJV shape is described in a qualitative way, as pinpoint, flattened, crescentic, and ellipsoidal [19].

In conclusion, the proposed procedure for the measurement and normalization of IJV CSA on healthy subjects is promising since it is a highly repeatable method, which allows the definition of a range of normality and group comparisons, and could also be applied to MRAV images.

## Figures and Tables

**Table 1:** Group average and standard deviation of IJVl and IJVr CSAs for all cervical levels between C1 and C7 considered separately and aggregated (TOT).

	IJVl CSA	IJVr CSA
	Mean (Std) (mm^2^)	Mean (Std) (mm^2^)
C1	41.87 (19.32)	52.70 (24.49)
C2	37.47 (19.00)	45.03 (21.86)
C3	45.29 (19.88)	54.10 (21.75)
C4	54.08 (18.51)	63.15 (23.99)
C5	62.19 (21.27)	71.71 (28.87)
C6	66.62 (25.78)	75.25 (31.88)
C7	66.92 (31.87)	79.98 (42.39)

TOT^*∗*^	53.52 (25.58)	63.26 (31.51)

^*∗*^Significant difference; *p* = 0.06.

**Table 2:** Intrarater and interraters variability of IJVl and IJVr CSA for all cervical levels from C1 to C7. The concordance correlation coefficient (*ρ*c), expressed also as precision (*ρ*) and accuracy (Cb), is reported. The intraclass correlation coefficient (ICC) is represented with its 95% lower (ci l) and upper (ci u) confidence interval bounds. Data shows almost perfect agreement (*ρ*c > 0.90) and no systematic differences (ICC > 0.9) between raters at each cervical level.

			C1	C2	C3	C4	C5	C6	C7
Intrarater	IJVl CSA	*ρ*c	0.989	0.989	0.982	0.907	0.961	0.971	0.960
		*ρ*	0.994	0.990	0.983	0.912	0.967	0.978	0.966
		Cb	0.995	0.999	0.999	0.995	0.994	0.993	0.994
		ICC	0.990	0.990	0.984	0.917	0.965	0.974	0.964
		95% ci l	0.960	0.956	0.934	0.686	0.857	0.891	0.859
		95% ci u	0.998	0.998	0.996	0.980	0.992	0.994	0.992
	IJVr CSA	*ρ*c	0.988	0.983	0.978	0.977	0.971	0.986	0.978
		*ρ*	0.989	0.984	0.983	0.979	0.979	0.987	0.989
		Cb	0.998	0.998	0.994	0.998	0.992	0.999	0.988
		ICC	0.989	0.984	0.980	0.979	0.974	0.987	0.980
		95% ci l	0.958	0.942	0.920	0.923	0.899	0.954	0.923
		95% ci u	0.997	0.996	0.995	0.995	0.994	0.997	0.995

Interraters	IJVl CSA	*ρ*c	0.969	0.992	0.994	0.975	0.964	0.955	0.934
		*ρ*	0.986	0.996	0.997	0.979	0.971	0.964	0.963
		Cb	0.983	0.996	0.997	0.996	0.993	0.991	0.969
		ICC	0.973	0.993	0.995	0.978	0.968	0.960	0.941
		95% ci l	0.770	0.972	0.970	0.911	0.873	0.843	0.763
		95% ci u	0.995	0.998	0.999	0.995	0.993	0.991	0.986
	IJVr CSA	*ρ*c	0.983	0.985	0.952	0.966	0.979	0.974	0.983
		*ρ*	0.992	0.987	0.969	0.977	0.985	0.988	0.988
		Cb	0.991	0.998	0.983	0.989	0.995	0.986	0.995
		ICC	0.985	0.986	0.957	0.969	0.981	0.977	0.985
		95% ci l	0.892	0.948	0.844	0.886	0.931	0.910	0.940
		95% ci u	0.997	0.997	0.989	0.992	0.995	0.994	0.996

**Table 3:** Intrarater and interraters Dice similarity coefficient (DSC) expressed as median (range).

	DSC IJVl CSA	DSC IJVr CSA
Intrarater	0.95 (0.78–1.00)	0.94 (0.77–1.00)
Interraters	0.95 (0.57–1.00)	0.94 (0.70–1.00)
